# Consensus-based technical recommendations for clinical translation of renal BOLD MRI

**DOI:** 10.1007/s10334-019-00802-x

**Published:** 2019-11-25

**Authors:** Octavia Bane, Iosif A. Mendichovszky, Bastien Milani, Ilona A. Dekkers, Jean-Francois Deux, Per Eckerbom, Nicolas Grenier, Michael E. Hall, Tsutomu Inoue, Christoffer Laustsen, Lilach O. Lerman, Chunlei Liu, Glen Morrell, Michael Pedersen, Menno Pruijm, Elizabeth A. Sadowski, Erdmann Seeliger, Kanishka Sharma, Harriet Thoeny, Peter Vermathen, Zhen J. Wang, Zbigniew Serafin, Jeff L. Zhang, Susan T. Francis, Steven Sourbron, Andreas Pohlmann, Sean B. Fain, Pottumarthi V. Prasad

**Affiliations:** 1 BioMedical Engineering and Imaging Institute and Department of Radiology, Icahn School of Medicine at Mount Sinai, New York, NY USA; 2 Department of Radiology, Cambridge University Hospitals NHS Foundation Trust, Cambridge University Hospitals NHS Foundation Trust, Addenbrooke’s Hospital, Cambridge, UK; 3 Center for BioMedical Imaging, Lausanne University Hospital and University of Lausanne, Lausanne, Switzerland; 4 Department of Radiology, Leiden University Medical Center, Leiden, The Netherlands; 5 Department of Radiology, Groupe Hospitalier Henri Mondor, Créteil, France; 6 Department of Radiology, Institution for Surgical Sciences, Uppsala University, Uppsala, Sweden; 7 Department of Radiology, Université de Bordeaux, CHU de Bordeaux, Bordeaux, France; 8 Department of Medicine, University of Mississippi Medical Center, Jackson, MS USA; 9 Department of Nephrology, Faculty of Medicine, Saitama Medical University, Saitama, Japan; 10 The MR Research Center Department of Clinical Medicine, Aarhus University, Aarhus, Denmark; 11 Division of Nephrology and Hypertension, Department of Internal Medicine, Mayo Clinic, Rochester, MN USA; 12 Electrical Engineering and Computer Science, and Helen Wills Neuroscience Institute, University of California Berkeley, Berkeley, CA USA; 13 Department of Radiology and Imaging Sciences, University of Utah, Salt Lake City, UT USA; 14 Department of Clinical Medicine-Comparative Medicine Lab, Aarhus University Hospital, Aarhus, Denmark; 15 Nephrology and Hypertension Service, Lausanne University Hospital and University of Lausanne, Lausanne, Switzerland; 16 Department of Radiology, University of Wisconsin School of Medicine and Public Health, Madison, WI USA; 17 Institute of Physiology, Charité–University Medicine Berlin, Berlin, Germany; 18 Imaging Biomarkers Group, Department of Biomedical Imaging Sciences, University of Leeds, Leeds, UK; 19 Department of Radiology, Hôpital Cantonal Fribourgois, University of Fribourg, Fribourg, Switzerland; 20 Departments for BioMedical Research and Radiology, Inselspital, Universitaetspital Bern, Bern, Switzerland; 21 Department of Radiology and Biomedical Imaging, University of California San Francisco Medical Center, San Francisco, CA USA; 22 Department of Radiology, Nicolaus Copernicus University, Collegium Medicum, Bydgoszcz, Poland; 23 Athinoula A. Martinos Center for Biomedical Imaging, Massachusetts General Hospital, Harvard Medical School, Boston, MA USA; 24 Sir Peter Mansfield Centre, University of Notthingham, Notthingham, UK; 25 Berlin Ultrahigh Field Facility, Max Delbrueck Center for Molecular Medicine in the Helmholtz Association, Berlin, Germany; 26 Departments of Biomedical Engineering, Radiology, and Medical Physics, University of Wisconsin, Madison, WI USA; 27 Department of Radiology, Center for Advanced Imaging, NorthShore University Health System, Evanston, IL USA

**Keywords:** BOLD MRI, Kidney, Imaging, Biomarkers, Standardization, Consensus

## Abstract

Harmonization of acquisition and analysis protocols is an important step in the validation of BOLD MRI as a renal biomarker. This harmonization initiative provides technical recommendations based on a consensus report with the aim to move towards standardized protocols that facilitate clinical translation and comparison of data across sites. We used a recently published systematic review paper, which included a detailed summary of renal BOLD MRI technical parameters and areas of investigation in its supplementary material, as the starting point in developing the survey questionnaires for seeking consensus. Survey data were collected via the Delphi consensus process from 24 researchers on renal BOLD MRI exam preparation, data acquisition, data analysis, and interpretation. Consensus was defined as ≥ 75% unanimity in response. Among 31 survey questions, 14 achieved consensus resolution, 12 showed clear respondent preference (65–74% agreement), and 5 showed equal (50/50%) split in opinion among respondents. Recommendations for subject preparation, data acquisition, processing and reporting are given based on the survey results and review of the literature. These technical recommendations are aimed towards increased inter-site harmonization, a first step towards standardization of renal BOLD MRI protocols across sites. We expect this to be an iterative process updated dynamically based on progress in the field.

## Introduction

Blood oxygenation level dependent (BOLD) MRI, as the name suggests, is an MRI contrast mechanism that depends on the oxygenation status of blood, specifically the oxygenation of blood hemoglobin (Hb) [1]. Oxygen saturation of Hb changes the magnetic properties of the Hb from being diamagnetic when fully oxygenated to paramagnetic in its deoxygenated state, which shortens the observed transverse free induction decay time constant, *T*_2_*, or lengthens the corresponding rate constant *R*_2_* = 1/*T*_2_*. Thus, a change in the ratio of oxy- to deoxy-Hb in blood results in a measurable change on BOLD MRI contrast and is widely used in functional MRI of the brain [2]. Since the ratio of oxy- to deoxy-Hb is a major determinant of blood partial pressure of oxygen (PO_2_), the method in principle can be used to measure an index of blood PO_2_. If one assumes that blood PO_2_ is in a dynamic equilibrium with the surrounding tissue PO_2_, then the BOLD MRI measurements can reflect tissue PO_2_. To reflect these underlying assumptions about the bio-distribution of PO_2_, the BOLD MRI *T*_2_* or *R*_2_* measure is sometimes referred to as “oxygen availability”. The method is completely non-invasive, since it utilizes an endogenous contrast mechanism and does not require injection of any exogenous contrast agent.

In contrast to virtually all other organs in the body, in the healthy kidney, not only the supply of oxygen, but also oxygen consumption is determined by blood flow [3]. The major determinant of oxygen consumption in the kidneys is sodium reabsorption. The amount of sodium to be reabsorbed is proportional to the glomerular filtration rate (GFR) which in turn, in general, changes in parallel with renal blood flow. Since both oxygen supply and demand are related to renal blood flow, evaluating renal oxygen status independent of perfusion is necessary in the kidneys. Much of the early data on renal oxygenation was obtained in animal models using invasive probes which have documented a gradient of decreasing oxygenation from cortex to inner medulla [4]. However, due to the lack of a non-invasive method for use in humans, very little was known about whether human kidneys have the same anatomical variations in oxygenation as observed in rodents. With the availability of BOLD MRI the presence of such a gradient was verified in the kidneys of healthy subjects [5]. Much of the early literature on BOLD MRI focused on the medulla, a region of naturally low oxygen tension. Since this region is known to be at risk for ischemic injury, understanding the endogenous mechanisms that maintain oxygenation status and effects of exogenous maneuvers that can modulate the oxygenation status were foci of early studies [6–10].

An early application of the BOLD MRI method to clinical studies was in renal artery stenosis where it was important to know who may benefit from re-vascularization [11]. Based on earlier reports in rodent models of diabetes [12, 13], there have also been studies assessing the relative oxygen status in individuals with diabetes and varying levels of renal function [14, 15]. With gaining interest in the chronic hypoxia hypothesis related to progressive chronic kidney disease (CKD) [16], there has been growing interest in evaluating renal oxygenation status in CKD [17–19] and kidney transplant [20–23]. This evolution towards clinical translation has led to the realization of the importance of standardization or harmonization of subject preparation, data acquisition and analysis methods. Without harmonization, it is currently difficult to objectively compare BOLD *T*_2_* (*R*_2_*) measures between sites and this constitutes a barrier for multi-center studies and pooling of clinically relevant data to inform future practice.

All physiological and functional imaging methods are inherently dependent on the physiological status of the organ at the time of data acquisition. To be able to compare data from one individual to another it is important to standardize the baseline physiological status (to the best extent possible). Water intake or hydration status has been shown to significantly influence renal oxygenation [6] as does salt intake [9]. Since consumption of food inherently involves consumption of water, fasting prior to the BOLD MRI study (e.g. 4 h) is commonly used. Controlling salt intake requires more regimented protocols and it is not practical to implement for routine clinical use.

**Fig. 1:**
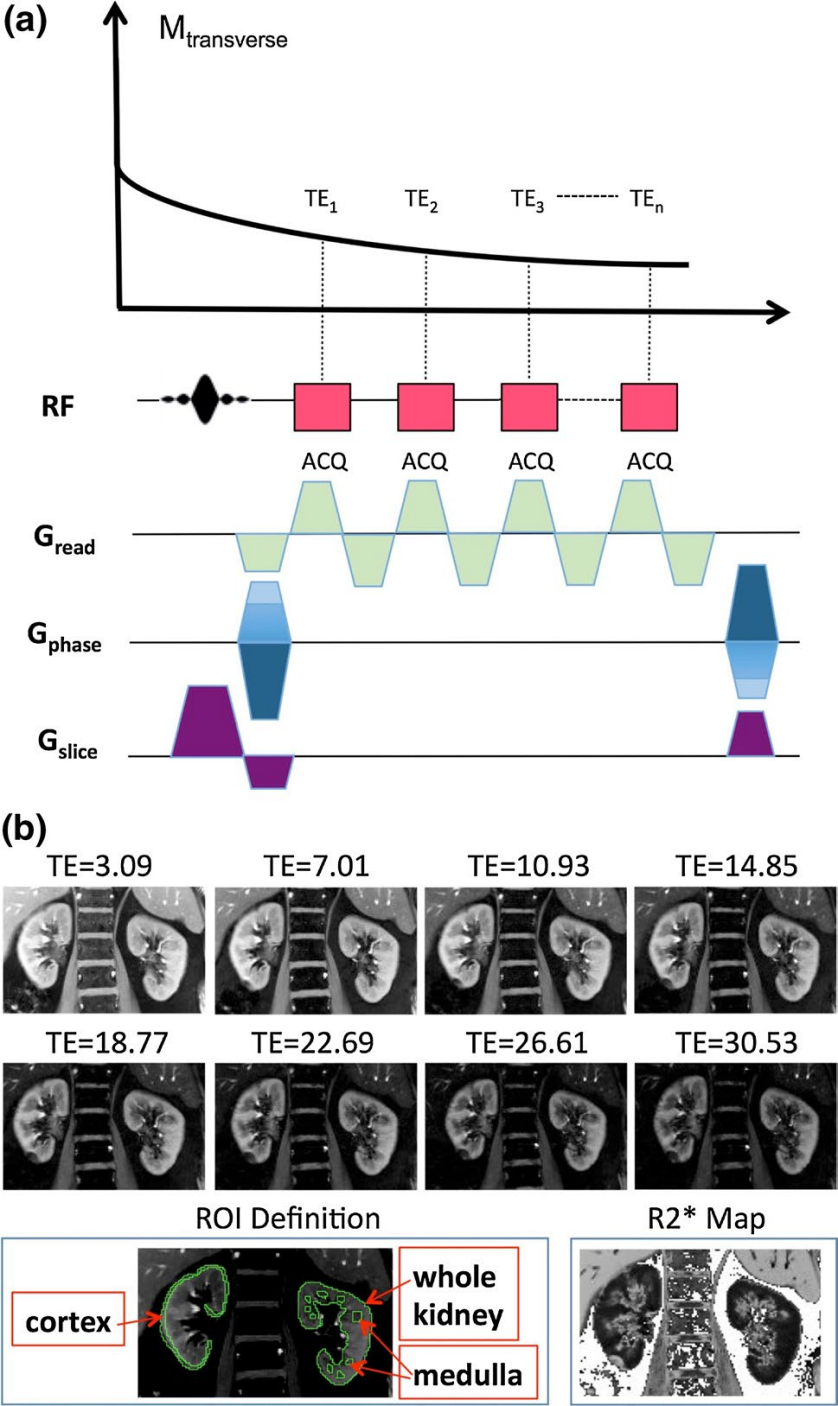
**a** Pulse sequence diagram of a multiple gradient-echo (mGRE) sequence with *n* echo times (TE_1_–TE_*n*_) obtained from a single RF excitation, with the readout gradient (*G*_read_) rewinded after each signal sampling (ACQ). **b** BOLD-MRI acquisition (breath hold, 2D mGRE sequence, TR = 51 ms, flip angle = 30**°**, 5 mm slice thickness, 5 mm space between slices, FOV 400 × 400 mm, matrix 256 × 256) example with eight echo times (TE from 3.09 to 30.53 ms, bandwidth 256 Hz/pixel); cortex and medulla ROI delineation example, and *R*_2_* grayscale map on a 3*T* Siemens Skyra^fit^ in a healthy volunteer (female, age 64, BMI 25.6). (Courtesy: Lu-Ping Li, Ph.D., NorthShore University Health System, Evanston, IL, USA). *BOLD* blood-oxygen level dependent, *FOV* field of view, *mGRE* multi-echo gradient echo, *RF* radio frequency, *ROI* region of interest, *TE* echo time

While early studies used single-shot echo planar imaging (EPI) sequences for renal BOLD MRI [5], the most widely used current method is the multiple gradient echo (mGRE) (Fig. 1a) acquisition [24, 25]. Typically 8–16 gradient echo images (Fig. 1) are acquired during breath-holds and a mono-exponential fit of the signal intensity with respect to echo time is used to estimate the relaxation time constant *T*_2_*. The sequence is widely available on all major vendor platforms and it typically comes with the option to calculate *T*_2_* or *R*_2_* online. While this has facilitated the widespread utility of the method, variations in the specific acquisition parameters make the comparison of data across sites difficult. Similarly, the subjective definition of manually defined regions of interest (ROIs) is widely reported and leads to an additional source of bias and variability, especially when related to the renal medulla (Fig. 1b). This problem of subjectivity is further aggravated in diseased kidneys since loss of cortico-medullary contrast is a hallmark in several forms of renal disease [26], including CKD. This has led to proposals to perform whole kidney analysis where the user only needs to manually trace the outer boundaries of renal parenchyma [19, 27, 28].

The objective for this initiative is to provide technical recommendations for renal BOLD MRI based on a consensus between experts in the field, aiming to promote a more standardized approach that will facilitate comparison of data across sites. We used a recently published statement paper [29], which included a detailed summary of parameters in their supplementary material, as the starting point to identify topics of interest in developing the questionnaires for seeking consensus. The work was facilitated by the ‘PARENCHIMA’ initiative “MRI Biomarkers for CKD” (CA16103), a community-driven Action of the European Cooperation in Science and Technology (COST) program of the European Union, which aims to improve the reproducibility and standardization of renal MRI biomarkers.

## Materials and methods

### Description of the survey process

During the PARENCHIMA Annual Plenary Meeting in Prague (October 4–5th 2018), the Task Force on Technical Recommendations for Clinical Renal MRI decided to follow a modified Delphi method [30–32] for seeking consensus regarding technical BOLD MRI acquisition and analysis. The Delphi method derives consensus recommendations through qualitative evaluation of published studies by experts. Where published data is scarce, experts can make inferences based on data from similar contexts, or their own experiences. Published evidence is summarized in the form of a questionnaire. Participants are invited to respond to the questionnaire in several rounds (typically 3), reviewing an anonymous summary of the previous responses before responding to the subsequent rounds. Respondents are asked to indicate whether they consider themselves sufficiently knowledgeable to answer each of the questions. Consensus on a question is usually defined as 68%-80% unanimity in responses [31–33]. Discussion in a face-to-face meeting between all respondents usually follows one or several of the rounds, and serves to build consensus in the areas where prior responses failed to achieve consensus. Owing to wide geographic distribution of the experts and respondents, we have adopted a modified Delphi method for this project with only a single face-to-face meeting before the final round of surveys.

### Construction of the questionnaire

The questionnaires were constructed by the panel co-chairs (PVP, OB and SF), between February 2019 and April 2019, and administered online to participants in two rounds *via* an online form (Google Forms, https://docs.google.com/forms). In constructing the questionnaires, the co-chairs used a recently published PARENCHIMA statement paper [29] and its detailed summary of parameters in the supplementary material, to formulate questions on renal BOLD MRI research topics, technical image acquisition parameters and analysis methods. Round 1 consisted of 35 questions with multiple-choice responses, with the possibility to abstain or to provide a long form answer. Participants were prompted to explain the reasoning behind their choices in a comment section following each item, and also to suggest questions for inclusion in Round 2 of the questionnaire.

Six members of the panel attended a face-to-face meeting between March 18th–19th 2019 at Aarhus University Hospital, Denmark. Based on a review of the first round of questionnaire responses, they worked together on additional questions and on the wording for specific questions in Round 2. Round 2 consisted of 31 items with answers in simplified multiple-choice format (“I agree”, “I disagree”, “I have insufficient experience to make a recommendation”) and possibility to explain the choice in a comment section following each item. Questions that reached consensus in Round 1 were summarized to the respondents in Round 2, and respondents were offered opportunity to comment if they did not agree. Respondents were encouraged to answer the questionnaire based on published evidence (some items pointed to specific publications) and best practices as reflected in the literature, not necessarily on their current research practice. Both iterations of the questionnaire had items grouped under four themes: (1) patient preparation (common items among all PARENCHIMA task force panels for Round 2, see covering review paper); (2) acquisition; (3) analysis; (4) application and interpretation.

### Panel selection

The first panel consisted of 10 PARENCHIMA task force members with a publication record and research interest in BOLD MRI. To expand our pool of respondents for the 2nd round, we invited other experts outside of our collaboration. We identified renal BOLD experts on the basis of a PUBMED literature search using [BOLD MRI] AND [kidney] AND [human]. An additional 16 respondents were enrolled while two respondents to the first questionnaire did not participate in the second one. For the second and final round of the questionnaire, we had 24 respondents from 22 academic institutions, 9 countries and 3 continents. All respondents are listed as authors of this manuscript. Panelists were asked to list their specialty when responding to the questionnaire.

### Interpretation of the survey results

At the Aarhus meeting, a “traffic light” system was adopted to issue recommendations based on the degree of consensus achieved through the questionnaire. Green light (consensus, closed issues) on a topic was defined as at least 75% unanimity in responses to a question. An “orange light” was defined in the case where the responses showed clear opinions (65–74%), but did not meet the threshold. For a “red light” topic, the questionnaire showed no clear expert preference (50–64%), so that no recommendation is possible with the information given to the respondents. It was also agreed that when calculating percentage of responses, the “abstain” responses (i.e. “I have insufficient experience to make a recommendation”) will be excluded. However, the percentage of “abstain” responses for each item will be reported, to reflect the level of familiarity of the experts with the topic and to show the topic’s importance (or lack thereof) in the clinical renal BOLD MRI community.

## Results

### Results of survey

The final results of the survey are summarized for each area in Tables 1, 2, 3, 4, 5, 6 and 7. For the second and final round of the survey, physicists (MR physicists or biomedical engineers) and clinicians (radiologists, nephrologists or physiologists) were equally represented (Table 2). The panelists reached consensus on 14 out of 31 items (45%) (Tables 3, 4, 5, 6). Additionally, panelists concurred on the nine questions for which consensus was achieved in Round 1 (Table 7). 12 items met the “orange light” definition, showing a clear direction of respondents’ opinions, while 5 items were “red light” topics with a 50/50% split in respondents’ opinions. Even though the overall response did not reach the 75% threshold, some questions had responses that reached the threshold in one of the sub-group analysis, Physicists or Clinicians. The presentation of responses by sub-group may be useful for our readers to draw perspectives, and so we have included sub-group results in all our tables.

**Table 1:** Final recommendations on renal BOLD MRI data acquisition, analysis and interpretation

	BOLD MRI
Preparation	**Normal hydration (100** **ml water)**, 4 h fasting from food
Field strength	**1.5 T or 3.0 T, 3*T*preferred if available**
Sequence	2D mGRE
Orientation	Coronal oblique to kidneys
In-plane resolution	**2–3** **mm**
Slice thickness	**3–5** **mm**
Coverage	3**–**5 slices centered on renal hilum
Parallel imaging factor	**2**
Fat suppression	Yes
TR (s)	60**–**75 ms
TE (ms)	**8–16 echoes, up to 50** **ms (~*T***_2_* cortex) at 3*T* with choice of in phase for fat-water
Averages	1
Breathing mode	**Breath hold**
Image quality control	Recommended
ROI placement	**Manual**
Cortical ROI	1 stripe/slice; > 3 slices
Medullary ROI	3 samples/slice; > 3 slices
Fitting	**Monoexponential or log-linear**
Reporting	**Cortex and medulla**
Reported metric	***R***_2_* (s^−**1**^**)**
Metric statistics reporting	Mean, median, standard deviation, ROI size
Map format	Color or grayscale quantitative map

Entries in bold font are based on the consensus process

*Bold* blood-oxygen level dependent, *ROI* region of interest, *T*_2_* transverse free-induction decay time, *TE* echo time

**Table 2:** Panel characteristics

Respondents for Round 1	*n* = 10	Percentage
Physicists	**7**	**70**
Clinicians	**3**	**30**
Radiologists	1	10
Nephrologists	2	20
Physiologists^a^	1	10

Respondents for Round 2	*n* = 24	Percentage
Physicists	**12 (50%)**	**50**
Clinicians/physiologists	**13 (50%)**	**54**
Radiologists^a^	11	46
Nephrologists	2	8
Physiologists^a^	1	4

The co-chairs sought equal representation of MR physicist and clinician researchers involved in BOLD MRI

^a^1 radiologist was also a Ph.D. physicist; 1 nephrologist was also a PhD physiologist

**Table 3:** Survey responses regarding patient preparation

	Consensus/tendency	Overall (%)			Physicists (%)			Clinicians (%)		
		Agree	Disagree	Abstain	Agree	Disagree	Abstain	Agree	Disagree	Abstain
1. Subjects’ diet needs to be controlled before the scan	Agree	71	29	13	60	40	17	**82**	18	15
**2. Subject should be scanned in a normal hydration status when clinically appropriate**	**AGREE**	**87**	13	4	**91**	9	8	**85**	15	0
3. Subjects are required to follow a controlled and standardized salt intake before the scan	Disagree	35	65	29	25	**75**	33	44	56	31

Agree/disagree responses are reported as percentage of all responses in each category, excluding abstain answers (“I have insufficient experience to make a recommendation”). Percentage of abstain responses is shown. Items that reached consensus are marked in capital letters and bold font

**Table 4:** Survey responses regarding image acquisition

	Consensus/tendency	Overall (%)			Physicists (%)			Clinicians		
		Agree	Disagree	Abstain	Agree	Disagree	Abstain	Agree	Disagree	Abstain
4. 3***T*** is better than 1.5T for acquiring quality BOLD data	Agree	74	26	21	75	25	33	73	27	15
5. The longest TE should be equal to the largest T_2_* expected in the kidney	Unresolved	53	47	38	55	45	8	40	60	62
6. The largest TE should be equal to 1.5 times the longest T_2_* expected in the kidney	Unresolved	53	47	38	55	45	8	60	40	62
7. Is the abdominal standard shim performed automatically by your scanner sufficient for quality BOLD data?	Disagree	35	65	17	36	64	8	30	70	23
**8. For quality BOLD data it is necessary to shim on a restricted volume (a “box” selected by the operator) that includes both kidneys**	**AGREE**	**76**	24	13	64	36	8	**91**	9	15
**9. For quality BOLD data it is necessary to shim on a restricted volume (a “box” selected by the operator) that includes only 1 kidney (accepting a poorer shim on the other kidney)**	**DISAGREE**	21	**79**	17	25	**75**	0	11	**78**	31
**10. Acquiring a 3D B0 map is important and useful for quality control of BOLD data**	**AGREE**	**80**	20	38	**86**	14	42	**67**	33	31
11. Reducing the effect of intra-renal fat improves BOLD data quality	Agree	69	31	33	**78**	22	25	50	50	38
12. For reducing the effect of fat, choosing fat and water to be in-phase for all echoes is preferable to using fat saturation	Agree	71	29	42	**78**	22	25	50	50	54
Most current reports on renal BOLD MRI have used 2 × 2 × 5 mm^3^ voxel size. There is evidence to suggest spatial resolution (i.e. voxel size) influences R_2_* estimates (PMID: 23571833). Please consider the evidence in this publication when responding to the following 4 questions, and keep your answers consistent										
13. Should BOLD acquisitions strive for a nearly isotropic resolution (e.g to help reduce unwanted effects of macroscopic field inhomogeneities)?	Agree	67	33	25	64	36	8	63	38	38
** 14. If you agree resolution should be isotropic, do you agree that 3 × 3× 3 mm**^3^** is a sufficient voxel size to balance SNR and resolution?**	**AGREE**	**79**	21	36	**86**	14	30	71	29	46
15. Considering your answer to the previous question, 2 × 2 × 5 mm^3^ is a sufficient voxel size for renal BOLD to balance SNR and resolution	Agree	65	35	29	56	44	25	**75**	25	38
**16. The right balance between parallel acceleration factor and SNR is a 2x acceleration factor for BOLD MRI**	**AGREE**	**93**	7	38	**100**	0	33	**86**	14	46

Agree/disagree responses are reported as percentage of all responses in each category, excluding abstain answers (“I have insufficient experience to make a recommendation”). Percentage of abstain responses is shown. Items that reached consensus are marked in bold font

*B0* main magnetic field, expressed in tesla (T), *BOLD* blood-oxygen level dependent, *SNR* signal-to-noise ratio, *T*_2_* transverse free-induction decay time, *TE* echo time

**Table 5:** Survey responses regarding data analysis

	Consensus/tendency	Overall (%)			Physicists (%)			Clinicians		
		Agree	Disagree	Abstain	Agree	Disagree	Abstain	Agree	Disagree	Abstain
**17. The marker of interest from renal BOLD is ***R*_2_*	**AGREE**	**100**	0	0	**100**	0	0	**100**	0	0
**18. Both exponential and log-linear fitting of the BOLD signal are acceptable for extracting ***R*_2_* **values**	**AGREE**	**83**	17	4	**75**	25	0	**83**	17	8
**19. Manual ROIs drawn on BOLD with anatomical T**_2_**or T**_1_**weighted images as reference, in collaboration with a radiologist, is a suitable analysis method for a novice user**	**AGREE**	**87**	13	4	**82**	18	8	**92**	8	8
20. Semi-automated segmentation of cortex and medulla (e.g. PMID: 28959212, using histogram analysis to define masks for cortex and medulla based on T_1_maps) is a preferred analysis method	Agree	72	28	25	70	30	17	**75**	25	38
**21. Semi-automated analysis which identified pixels with “hypoxia” based on a predetermined threshold ***R*_2_* **(PMID: 23788716) value within the kidney is a preferred analysis method**	**DISAGREE**	25	**75**	33	0	**100**	33	50	50	38
22. Histogram analysis of T _2_*/R_2_* maps is a preferred analysis method	Unresolved	50	50	42	25	**75**	33	83	17	54
23. The TLCO or onion peel (PMID: 27798200) is a preferred analysis method	Unresolved	53	47	38	71	29	42	38	63	38

Agree/disagree responses are reported as percentage of all responses in each category, excluding abstain answers (“I have insufficient experience to make a recommendation”). Percentage of abstain responses is shown. Items that reached consensus are marked in bold font

*BOLD* blood-oxygen level dependent, *R*_2_* transverse free induction decay rate constant, *T*_*1*_ longitudinal relaxation time, *TLCO* twelve-layer concentric objects (renal image segmentation method)

**Table 6:** Survey responses regarding BOLD application and data interpretation

	Consensus/tendency	Overall (%)			Physicists (%)			Clinicians (%)		
		Agree	Disagree	Abstain	Agree	Disagree	Abstain	Agree	Disagree	Abstain
**24. Given the consensus that *R***_2_* is a relative measure,*R*_2_* measures oxygen availability rather than absolute oxygenation (e.g. partial pressure of oxygen) in the kidney	**AGREE**	**87**	13	4	**82**	18	8	**85**	15	0
**25. Renal BOLD MRI is potentially a diagnostic technique**	**AGREE**	**78**	22	4	**64**	36	8	**92**	8	0
26. Renal BOLD MRI is currently only useful as a research tool	Agree	74	26	4	**75**	25	0	**75**	25	8
**27. The most proven application for renal BOLD MRI is to study acute responses to physiological or pharmacological maneuvers**	**AGREE**	**86**	14	13	**100**	0	17	**75**	25	8
28. From published data to-date, renal BOLD MRI can be used to evaluate acute kidney dysfunction (acute kidney injury, allograft acute rejection, allograft acute tubular necrosis)	Agree	67	33	25	**75**	25	33	60	40	23
29. From published data to-date, renal BOLD MRI can be used to evaluate chronic kidney dysfunction (interstitial fibrosis and tubular atrophy)	Unresolved	53	47	21	44	56	25	60	40	23
**30. From published data to-date, has renal BOLD MRI been shown to have sufficient (short and long term) scan-re-scan repeatability for translational research studies?**	**AGREE**	**85**	15	17	**100**	0	17	**70**	30	23
31. From published data to-date, has renal BOLD MRI been shown to have sufficient (short and long term) scan-re-scan repeatability for multi-site clinical trials?	Disagree	32	68	21	44	56	25	18	82	15

Agree/disagree responses are reported as percentage of all responses in each category, excluding abstain answers (“I have insufficient experience to make a recommendation”). Percentage of abstain responses is shown. Items that reached consensus are marked in bold font

*BOLD* blood-oxygen level dependent, *R*_2_* transverse free induction decay rate constant

**Table 7:** Survey items from Round 1 which achieved consensus, excluding abstain (“Do not know”) and “Other” answers

	Overall (%)
1.5*T* is acceptable for obtaining quality BOLD data. [choose a single option]	
Agree (comment in other)	100
Disagree (comment in other)	0
Do not know	30
Other	0
The smallest bandwidth should be chosen for the selected maximum TE	
Agree (comment in other)	83
Disagree (comment in other)	16
Do not know	30
Other	10
Do number of echoes and/or spacing between TE’s have an effect on data quality?	
Agree (comment in other)	88
Disagree (comment in other)	12
Do not know	20
Other	12.5
Is breath-holding the best approach for controlling motion in renal BOLD MRI?	
Yes	80
No	20
Do not know	0
Other	0
Is analysis of cortex and medulla important?	
Yes, both equally important	90
Cortex is more important than medulla (comment in other)	0
Medulla is more important than cortex (comment in other)	10
Do not know	0
Other	0
Is renal BOLD MRI potentially a prognostic technique?	
Yes	100
No	0
Do not know	20
Other	0
Does renal BOLD MRI reflect intra-renal oxygenation (qualitatively or quantitatively)?	
Yes, BOLD MRI can quantify intrarenal oxygenation	20
Yes, qualitatively, there are too many confounding factors for it to be quantitative	80
No, it does not	0
Do not know	0
Other	0
From existing data, do you believe renal BOLD MRI is more suited to detect changes in renal oxygenation within the same kidney rather than comparing cohorts?	
Yes, best used to detect changes within the same kidney, due to confounding effects of blood volume and hematocrit that are different between patients	88
It is suitable to compare cohorts without adjustments for patients’ blood volume and hematocrit	0
It is suitable to compare cohorts with adjustment for patients’ blood volume and hematocrit	11
Do not know	10
Other	0
Does the furosemide stress test/ physiological challenge have value?	
Yes, always	25
Yes, but it may not be necessary or suitable for some applications (comment in Other)	75
No (comment in Other)	0
Do not know	20
Other	0

These items and the dominant response percentages were shown to participants in Round 2, who concurred to the consensus in Round 1

*Bold *blood-oxygen level dependent, *TE* echo time

### Final recommendations

Our final recommendations, summarized in Table 1, are based on the literature, the consensus views and opinion trends that emerged from the survey and the face-to-face meeting.

- Regarding patient preparation, irrespective of the level of consensus reached, users should consider ways of normalizing the baseline physiological status. Even though data exists only for hydration status, food intake commonly is associated with fluid intake and a likely confounding factor. When feasible, fasting for 4 h may be a good option. A related issue of bowel gas has not yet been fully resolved in terms of minimizing its presence.
- Even though the choice of 3*T* did not reach the 75% threshold, there is a general acceptance that, if given a choice between the field strengths, 3*T* is preferable. In the absence of access to 3*T*, 1.5*T* remains an adequate choice. While there is a concern for increased magnetic field inhomogeneities at 3*T*, there is considerable experience to-date that supports the higher sensitivity and signal-to-noise ratio (SNR) afforded by 3*T*, making it a preferred choice.
- Magnetic field inhomogeneities (e.g. due to poor shimming, presence of metal or air interfaces) play an important role and should be minimized. These will affect the measured *R*_2_* values and results in a voxel-size dependence of *R*_2_*. Slice thickness is the largest contributor to voxel size, and has the largest contribution to the voxel-size dependence of *R*_2_* due to through-slice dephasing although the effects may be lower in the coronal orientation. While smaller and isotropic voxels are preferred, they are limited by the need for breath-holding. Adopting a fixed voxel size for all studies may be a preferred way to standardize the protocol and allow comparisons across sites. This is challenging to implement in routine practice because voxel size can be set directly on the scanners of one vendor (e.g. Philips), while in other vendors’ scanners (e.g. Siemens, GE) the voxel size is derived from field of view (FOV) and matrix size. Even if matrix size is kept constant, MRI technologists are inclined to change FOV based on the body habitus. Thus, standard operating procedures (SOPs) are necessary and the MRI technologist needs to be instructed not to change the FOV when prescribing renal BOLD MRI.
- The presence of fat may have an effect on measured *R*_2_* values, and use of fat saturation or water-excitation pulses is preferred.
- Most studies to-date have focused on the renal medulla which has a higher *R*_2_* (~30 s^−1^ at 3*T*) and hence a shorter *T*_2_* (~33 ms). In comparison, the renal cortex has *R*_2_* ~20 s^−1^ at 3*T* and *T*_2_*~50 ms. After fixing the longest TE = 50 ms, investigators need to decide the number of echo times. For a robust acquisition, any number of echoes between 8 and 16 would be acceptable. The actual number of echoes that is realizable will depend on readout bandwidth, echo spacing and image resolution. The bandwidth should be kept to around 300 Hz/pixel. With this echo time, use of either a mono-exponential or log-linear fit would be acceptable since the noise floor will not be reached.
- *R*_2_* was the preferred metric and there was consensus for evaluating both cortical and medullary regions. ROI analysis was preferred, only because of widespread availability. While newer custom methods have advantages such as better objectivity, these also have several limitations. Until automated segmentation becomes routinely available, manual segmentation may be preferred.
- There was an agreement that BOLD MRI provides a qualitative measure of relative oxygen availability and may be most suited for evaluating acute changes within the same kidney, e.g. following administration of furosemide. However, caution was advised when comparing different cohorts because of potential confounding effects such as blood volume fraction, oxy-Hb dissociation curve, and haematocrit [34]. Even though there was consensus in terms of reproducibility at a single site, similar data for multi-site studies remains lacking, and is thus desirable for future studies.
- Similar recommendations are made for BOLD in native kidneys and allografts. However, the allografts’ positioning in the iliac fossa, closer to the skin during an end-expiration breath-hold, sometimes requires different mitigation strategies for the tissue-air interface susceptibility artefacts (e.g. placing a bag of saline on the skin over the expected region of the allograft before placing the imaging coil on the subject). Planning of slices in true coronal (to the allograft) orientation is somewhat more difficult than with native kidneys and in some studies an oblique sagittal slice with respect to the allograft’ longitudinal axis is preferred.

## Discussion

### Patient preparation

Three identical items on patient preparation prior to the MRI scan (Table 3) were included in all PARENCHIMA panels’ questionnaires on clinical renal MRI. To-date, these questions are most relevant for the BOLD MRI panel. A previous systematic review conducted by PARENCHIMA [29] showed that of 20 studies on drug and dietary effects on renal BOLD MRI, 12 studies required their subjects to abstain from food and water for 12 h before MRI, with (8 studies) and without (4 studies) subsequent water loading, 1 study had a shorter fasting period of 2 h before MRI, and 7 studies did not require fasting. The variety of patient preparation in published studies was reflected in the responses to our survey. The respondents did not reach consensus on the need to control subjects’ diet before the scan, although 82% of clinicians were in favor of controlling the diet. Respondents’ comments to the question may explain the lack of consensus:

- 25% of respondents disagreed that diet should be controlled,
- 4% cited lack of publications showing an effect of diet on BOLD signal, and
- 4% thought that monitoring and reporting subjects’ diet is sufficient.
- 12.5% of respondents recommended fasting (water only intake) for 4–6 h before the scan, 8% went further and recommended abstaining from high protein foods 24 h before the scan in addition to fasting, to minimize susceptibility artifacts from bowel gas.
- Another 8% of respondents thought restricting drinking before the scan can reduce artifacts from the bowel, and can have an effect on BOLD quality.
- Another 8% of respondents recommended controlling sodium intake, possibly through standardized meals for study subjects in prospective studies.

The respondents reached consensus (87%) that patients should be scanned in a normal (i.e. not restricted to water intake) hydration status, based on studies that show the influence of hydration status on BOLD signal (as mentioned by 8% of respondents). Respondents agreed that hydration status should be controlled, through fasting (4%) or standardized (i.e. all subjects treated similarly in terms of hydration [35]) intake of water (8%).

There was no consensus that sodium intake should be standardized before the scan. 65% of respondents tended to disagree. Even though disagreement among physicists reached consensus threshold (75%), there was a split opinion among clinicians (agree/disagree: 44%/56%). Eight percent of the respondents mentioned their awareness of studies showing that salt intake influences BOLD measurements, with the caveat that the influence of sodium and hydration on the BOLD signal is smaller in the cortex than in the medulla. Modifying salt intake also influences kidney physiology, and may thus confound studies looking at subjects’ typical renal physiology, as commented by another 8% of respondents. 12.5% of respondents motivated their disagreement to this item by the practical difficulties of controlling subjects’ sodium intake, as salt is found in most processed foods. 8% of respondents recommended that controlled sodium intake is feasible for in-patient subjects who can follow a standardized diet during their hospital stay. While standardizing salt intake is not possible or suitable for the study design, monitoring and recording salt intake (e.g. via food diaries) by the subjects can add to any one study’s rigor.

### Data acquisition

The results of the survey items on data acquisition are summarized in Tables 4 and 7. We sought expert opinion on aspects of BOLD MRI data acquisition protocol including choice of field strength, reduction of B0 inhomogeneities, reducing the effect of intra-renal fat, choice of spatial resolution, magnitude and number of echo times (TE) and control of respiratory motion.

Renal BOLD MRI in humans has been performed at both 1.5 and 3*T* in almost equal distribution to-date: of 79 renal BOLD MRI human and animal studies included in a previous PARENCHIMA systematic review, 41 were performed at 1.5*T*, 42 at 3*T*, and four used magnets of both field strengths [29]. Even though there was consensus (Table 7; 100% of non-abstaining respondents, with 30% abstention rate) that 1.5*T* provides BOLD data of acceptable quality, there was a general preference for 3*T* over 1.5*T* (Table 4) (74% agreement). 3*T* has the known advantage of greater SNR, increased *T*_2_* weighting and hence higher *R*_2_* values. However, magnetic field inhomogeneities, bulk magnetic susceptibility (BMS) artifacts and fat-water chemical shift are also magnified at 3*T* [29]. Twenty percent of respondents mentioned these disadvantages of 3*T* in imaging the kidney near complex abdominal structures like the bowel, when motivating their disagreement or hesitancy to agree with the statement that 3*T* was superior. For control of respiratory motion, consensus was reached already in Round 1 (Table 7) that breath-holding is the best approach for both native kidneys and transplants (80%), although panelists who disagreed (20%) were familiar with or working on respiratory-triggered sequences.

There was consensus in Round 1 in choosing the smallest receiver bandwidth for the selected maximum TE (and number of echoes) in order to maximize SNR, and most respondents (88%) thought that the number of echoes and spacing between TEs have an effect on data quality (Table 7). It was generally agreed that more acquired TEs can improve robustness of *T*_2_*/*R*_2_* fitting. Eight to sixteen echoes are commonly used in the literature to-date. Expert opinion was evenly split regarding choice of longest TE based on the longest *T*_2_* expected in the kidney (Table 4). While 53% of non-abstaining respondents tended to agree that the longest TE should be equal to the longest *T*_2_* there were concerns that it may be too conservative (e.g. more data can be acquired beyond that value to improve the fit). Conversely, acquiring up to 1.5 times the longest *T*_2_* expected in renal tissue, while preferable to 53% of respondents, raised concerns that the noise floor could be reached in tissues with low oxygenation, that the TR, and hence breath-hold interval, would be increased too much, or that good image quality could not be obtained with some coils at TE = 1.5 times longest *T*_2_* expected in the kidney.

With regards to mitigating (or minimizing) B_0_ inhomogeneities, most respondents (65%, Table 4) did not consider the standard abdominal shim performed by the scanner to be sufficient for quality BOLD data. There was overall consensus that a restricted shim should be performed on a volume encompassing both kidneys (76% of all respondents, 91% of clinicians, but only 64% of physicists, agreed), but not on a smaller volume encompassing each kidney separately (79% of all respondents, and 75% of physicists and 78% of clinicians, disagreed). The main concern with shimming on each kidney separately was the time consuming workflow (12.5% of respondents), strongly localized shimming introducing artifacts from outside the shimmed area (8% of respondents), while 1 respondent observed no difference in *T*_2_* maps between the kidneys when shimming for each kidney separately. Respondents using BOLD in the study of kidney transplants used saline bags placed over the skin at the location of the allograft to mitigate susceptibility artifacts from the allograft’s proximity to the body surface and from bowel gas [21, 23]. Respondents acknowledged that localized shimming could not always address B_0_ inhomogeneities, which is why there was consensus that acquiring a 3D B_0_ map is useful for quality control of BOLD data (80% of non-abstaining respondents, 78% of physicists, and 67% of clinicians agreed). Acquisition of many BOLD datasets with B_0_ maps should also foster the development of widely available open-source post-processing software tools that correct the measured *R*_2_* by removing the effect of B_0_ inhomogeneity facilitating multicentre studies. Among physicians 67% of non-abstaining respondents agreed on the question of the choice of B_0_ map, and there was a high rate of abstaining answers (31–42%), which suggests a low availability or utilization of B_0_ mapping sequences and techniques on clinical scanners. However, a dual-echo GRE scheme to generate B_0_ maps from the phase difference of the two echoes is now available on all MR vendors. It should be noted that there are no reports in the published literature yet to support these recommendations regarding shimming, which may explain the high rate of abstain responses.

One manifestation of B_0_ inhomogeneity is the voxel-size dependence of *R*_2_* estimates [36]. There was evidence to show that slice thickness (usually larger than in-plane voxel size) contributed more to the observed effect [36]. The panelists were asked to consider this evidence [36] when responding to the last four items of the Acquisition portion of the survey (Table 4). While consensus was not achieved on the need for a nearly isotropic resolution, a sizeable portion of non-abstaining respondents (67%) thought isotropic voxels were preferred for imaging an organ with complex structures like the kidney. However, isotropic imaging raised concerns because of the need to match voxels in multi-parametric protocols with other MRI contrasts that are not best suited to an isotropic resolution protocol, such as arterial spin labeling (ASL). There was consensus for using 3 × 3 mm^2^ in-plane resolution (not necessarily isotropic). Similarly consensus was reached for using acceleration factor of 2 to achieve anatomical coverage within a breath-hold while maintaining sufficient SNR.

Studies in the liver demonstrate that the presence of fat can influence *T*_2_*/*R*_2_* measurements [37]. While healthy kidneys do not contain intra-renal fat, in some diseased states such as diabetes [38] and obesity [39], there may be intra-renal fat present. For this and other reasons (delineation of kidney), fat saturation/water excitation is desirable for BOLD MRI acquisitions, but not widely used. The low utilization of these techniques is reflected in the response to the question of whether it is preferable to control for the presence of fat by selection of all echo times with fat-water in phase rather than by performing fat saturation (71% agreement, 42% abstain rate). The statement reached agreement among physicists (78%), but not among clinicians (50/50% split, with 38–54% abstain rate). Because of the lack of consensus, but tendency towards a choice of in-phase fat-water TEs, we recommend performing both fat saturation/suppression and choosing in-phase TEs.

### Data analysis

The results of the survey items on data analysis are summarized in Tables 5 and 7. 100% of respondents agreed that the reported value should be *R*_2_* (rather than *T*_2_*) obtained from exponential or log-linear fit of BOLD signal (83%). However, respondents cautioned that the results are not comparable between fitting methods, and that the log-linear fit increases the influence of noise for later echoes with low SNR. A possible solution to correcting for noise at later echoes is to use a weighted log-linear fit. An exponential fit should also be corrected for noise, after identifying the noise floor. Of course, this concern can be mitigated by limiting the maximum TE to be ~ *T*_2_* of tissue of interest.

In Round 1, respondents reached consensus that measuring *R*_2_* values in both the cortex and medulla is equally important (Table 7; 90%). Early renal BOLD MRI research was driven by the low oxygenation and greater sensitivity to changes in oxygen demand in the medulla. Some studies recommended the medulla-to-cortex ratio in *R*_2_* to be a more sensitive marker especially in diabetes, taking advantage of the disparate changes with disease severity in cortex vs. medulla [14]. Increased *R*_2_* in the cortex has been observed in chronic kidney disease (CKD) [19], with either minimal change or actually a reduction in medullary *R*_2_* [15, 40, 41]. The consensus among all respondents (Table 5; 82%) was that a novice BOLD researcher can obtain *R*_2_* measurements in both cortex and medulla using regions of interest (ROIs) drawn in collaboration with a radiologist. *R*_2_* measurements in this case can be based on average signal in the ROI (for protocol optimization), or pixel-based within the ROIs. Ideally, manual ROIs should delineate as much of the cortex and medulla as possible, avoiding lesions, large vessels and fat.

More than with image acquisition, there was greater difficulty in obtaining consensus on advanced, semi-automatic image analysis methods. This is due to the lack of availability and experience with each method beyond the proponent site. Among advanced methods of ROI segmentation, semi-automated segmentation of cortex and medulla using histogram analysis to define masks for cortex and medulla based on *T*_1_ maps [42] nearly reached consensus among 72% of respondents (75% of clinicians, 70% of physicists). Histogram analysis of *T*_2_*/*R*_2_* maps and the 12-layer concentric objects (TLCO) or “onion peel” method [19] generated a 50/50 split in preferences among respondents. There was agreement among physicists (75%), but not among clinicians (50–50% split of opinion) that semi-automated analysis methods, which identify pixels as being “hypoxic” based on a predetermined threshold value for *R*_2_* [28], are not preferred. Concerns regarding implementation, as it is difficult (if not impossible, given the effects of shimming) to decide on a reliable threshold for hypoxia for the condition or patient population studied. Some common concerns expressed by respondents regarding semi-automated segmentation methods were that segmentation software packages are not widely available, have been tested only with particular acquisition protocols (e.g. preference for true coronal to the kidney orientation for the TLCO method [19]), may not work so well in diseased kidneys with poor cortico-medullary differentiation, and have not been validated across sites.

### Application and interpretation

The results of the survey items on BOLD applications and data interpretation are summarized in Tables 6 and 7. In Round 1 (Table 7), consensus (80%) was achieved among respondents that renal BOLD MRI can reflect oxygenation qualitatively, as the confounding factors of perfusion, blood volume fraction (per tissue volume), hematocrit, and shifts of the oxy-Hb dissociation curve preclude it from being truly quantitative. For that reason, respondents consented to the statements that renal BOLD MRI is more suited to detect changes in renal oxygenation within the same kidney rather than for comparing cohorts (88%), and that examining changes in *R*_2_* brought on by a furosemide challenge is useful, although not necessary for all applications (75%).

The consensus statement emerging from Round 1 that BOLD MRI provides a relative measure of oxygen availability, rather than an absolute measure of oxygenation, was supported in Round 2 (Table 6; 87% of respondents). The consensus from Round 1 that BOLD MRI is a potentially prognostic technique (100% non-abstaining respondents) was further improved in Round 2, with 78% of respondents (Table 6) considering BOLD a potentially diagnostic technique. There was notable split among clinicians (92%) and physicists (63%) on the diagnostic value of BOLD. The statement that BOLD MRI is currently only useful as a research tool (Table 6) narrowly missed the consensus threshold with 74% overall. Respondents commented that BOLD can only be diagnostic in combination with other methods, due to the confounding factors, and that its clinical impact has to be better demonstrated beyond a few experienced centers.

Among applications of BOLD MRI, the consensus (Table 6; 86% overall) emerged that renal BOLD MRI is best suited for studying acute responses to physiological or pharmacological maneuvers (e.g. water or furosemide challenge). Fewer respondents considered renal BOLD suitable for evaluation of acute dysfunction in native kidneys or allografts (67% overall, 75% of physicists, 60% of clinicians) or chronic renal dysfunction (53% overall, 44% physicists, 60% clinicians). Although there are studies showing differences between groups in acute dysfunction [43, 44], respondents (8%) commented there are better ways to assess renal dysfunction, and that more work is needed to show clinical relevance of BOLD MRI (8%). The division in opinion on the utility of BOLD in CKD is based on the published studies, which found increased *R*_2_* (lower oxygenation) [14, 45] or decreased *R*_2_* [15] in patients with CKD compared to controls, and did not show a significant correlation of *R*_2_* with estimated GFR or CKD stage [35, 40, 46].

With regards to short and long-term scan-rescan repeatability, the consensus among respondents (85%) was that the literature [47, 48] shows high repeatability for translational research, within the same site, if shimming and physiological conditions (diet, hydration) are controlled. However, 68% of respondents disagreed that multi-center repeatability suitable for clinical trials has been shown in the literature to-date. Respondents commented that standardization of protocols and more accumulation of results from multi-center studies is needed.

### Issues reaching consensus

Most items that did not reach consensus are related to split opinions in the literature, or limited data available to-date. The fact that our respondents did not reach consensus on superiority of imaging at 3*T*, standardizing diet, or salt intake reflects the even split among published studies: studies at 1.5*T* and 3*T* are equally represented in the literature, and a minority of published studies have standardized salt intake [29]. Split opinion on the longest TE is also due to a lack of systematic comparison studies exploring how feasible it is to image at long TE’s (1× or 1.5 x *T*_2_* of cortex) on different platforms, with respondents relying on their individual experience. More than with image acquisition, there was greatest difficulty in obtaining consensus on advanced, semi-automatic image analysis methods. This is due to the lack of availability and experience with each method beyond the proponent site.

### Limitations

The panel of experts that participated in this consensus formation process was of a relatively small size (*n * = 24). However, it included an equal proportion of clinical and technically oriented scientists from groups that have all developed or applied renal BOLD MRI. Given the small sample size, we did not record other factors that may influence responses, such as respondents’ age, years of experience in BOLD MRI, and location by country. In future recommendation surveys and papers, we plan to account for how these factors influence the recommendations. Although consensus emerged for acquisition methods, the greatest variability of responses covered analysis and interpretation of data, particularly semi-automatic segmentation of BOLD MRI cortical and medullary data, since only a limited number of dedicated research groups have experience with the advanced analysis methods.

### Remaining challenges for future research

The unresolved issues emerging from this consensus process point to potential avenues for future research. More systematic studies are needed on whether controlling diet beyond a 4-h fast before the scan through standardized diet and salt intake has an effect on renal BOLD MRI. Technical development, validation and dissemination is needed for B_0_ mapping and related methods to mitigate B_0_ inhomogeneities and bulk susceptibility artifacts. Similarly, respiratory motion control by breath-holding is a limiting factor, creating a trade-off between the number of echo times or slices acquired, which can be addressed by further development of fast free-breathing BOLD MRI sequences or respiratory motion correction methods. Respiratory motion control beyond breath-holding is essential for achieving whole kidney coverage in BOLD MRI acquisitions.

On the data analysis side, the survey responses underline the need for dedicated renal BOLD MRI post-processing software to facilitate semi-automated image analysis, with validation and dissemination of the software beyond the most experienced sites. The development and validation of (semi-)automatic segmentations methods for renal BOLD MRI data should be encouraged, especially in patient populations with poor cortico-medullary differentiation, where manual selection of cortical and medullary ROIs can be biased.

There is a future role for artificial neural networks/ artificial intelligence (AI) in solving cortex and medulla segmentation problems on BOLD MRI data acquired in patients with renal disease. To date, there are no published studies on AI in renal BOLD MRI, but artificial neural networks have been used in the brain for quantitative analysis of BOLD MRI and quantitative susceptibility mapping data [49]. AI methods have been used for automatic segmentation of the cortex and medulla for renal volumetry based on *T*_1_-weighted imaging, with [50] and without gadolinium contrast enhancement [51–53]. The ROIs obtained by automatic segmentation of T_1_-weighted images can inform segmentation of the cortex and medulla in a multiparametric MRI protocol. We plan to include a literature review as the field develops, and survey questions on AI in renal BOLD MRI in future iterations of the recommendations. Use of AI requires large training sets of manually annotated, high-quality renal BOLD MRI data, with robust clinical validation and correlation with patient-centric outcomes [54]. Increased quality and standardization in data acquisition is thus needed for the large-scale deployment of AI in renal BOLD MRI. Multicenter studies with harmonized scanning protocols, uniform semi-automatic data analysis, and the development of a phantom with reference cortical and medullary *R*_2_* values are high priority for the qualification of BOLD MRI *R*_2_* as a renal biomarker. Additional challenges are also likely to emerge with better understanding of the kidney’s complex physiology and pathophysiology, such as the degree to which renal functional markers are influenced by the huge range of drugs used to treat renal and systemic diseases, as well as how these measured renal parameters change in multi-morbidity states.

## Conclusion

This collaborative effort was undertaken with the aim of putting forward technical recommendations for facilitating the more widespread use of renal BOLD MRI protocols for clinical research and hopefully their eventual translation to the clinical setting. Based on the current literature and using a modified Delphi method to seek consensus, we have developed a set of recommendations for renal BOLD MRI data collection. The recommendations are to be considered as guidelines to motivate translation and multi-center studies. Given the dynamic nature of physiological imaging methods, techniques are bound to evolve, especially in terms of data acquisition and analysis of renal BOLD MRI. Therefore, we expect and encourage periodic updates of these recommendations.
